# Evaluating swine disease occurrence on farms using the state-space model based on meat inspection data: a time-series analysis

**DOI:** 10.1186/s40813-024-00355-z

**Published:** 2024-01-23

**Authors:** Tsubasa Narita, Meiko Kubo, Yuichi Nagakura, Satoshi Sekiguchi

**Affiliations:** 1 Graduate School of Medicine and Veterinary Medicine, University of Miyazaki, Miyazaki, 889-1692 Japan; 2 Miyazaki Prefectural Institute for Public Health and Environment, Miyazaki, 889-2155 Japan; 3 Miyazaki Prefectural Takasaki Meat Inspection Center, Miyazaki, 889-4505 Japan; 4 Miyazaki Prefectural Miyakonojo Meat Inspection Center, Miyazaki, 885-0021 Japan; 5 Department of Veterinary Science, Faculty of Agriculture, University of Miyazaki, 1-1, Gakuen-Kibanadai-Nishi, Miyazaki-Shi, Miyazaki Prefecture 889-2192 Japan; 6 Center for Animal Disease Control, University of Miyazaki, Miyazaki, 889-2192 Japan

**Keywords:** Swine, Slaughterhouse, Inspection data, State-space model

## Abstract

**Background:**

Data on abnormal health conditions in animals obtained from slaughter inspection are important for identifying problems in fattening management. However, methods to objectively evaluate diseases on farms using inspection data has not yet been well established. It is important to assess fattening management on farms using data obtained from slaughter inspection. In this study, we developed the state-space model to evaluate swine morbidity using slaughter inspection data.

**Results:**

The most appropriate model for each disease was constructed using the state-space model. Data on 11 diseases in slaughterhouses over the past 4 years were used to build the model. The model was validated using data from 14 farms. The local-level model (the simplest model) was the best model for all diseases. We found that the analysis of slaughter data using the state-space model could construct a model with greater accuracy and flexibility than the ARIMA model. In this study, no seasonality or trend model was selected for any disease. It is thought that models with seasonality were not selected because diseases in swine shipped to slaughterhouses were the result of illness at some point during the 6-month fattening period between birth and shipment.

**Conclusion:**

Evaluation of previous diseases helps with the objective understanding of problems in fattening management. We believe that clarifying how farms manage fattening of their pigs will lead to improved farm profits. In that respect, it is important to use slaughterhouse data for fattening evaluation, and it is extremely useful to use mathematical models for slaughterhouse data. However, in this research, the model was constructed on the assumption of normality and linearity. In the future, we believe that we can build a more accurate model by considering models that assume non-normality and non-linearity.

**Supplementary Information:**

The online version contains supplementary material available at 10.1186/s40813-024-00355-z.

## Background

Slaughter inspection is performed to confirm the safety of meat. In many countries, livestock that are slaughtered for food are required by law to be inspected at meat inspection centers. Slaughter inspection consists of two processes: the ante-mortem inspection to inspect live animals, and the post-mortem inspection to check for abnormalities in internal organs and carcasses. To identify abnormal health conditions, meat inspection centers conduct microbiological, pathological, and physicochemical examinations in addition to visual and palpation inspections. The inspection information of animals assessed by inspectors is recorded for each farm and is returned to the farms to help in preventing animal diseases and enhance the safety of meat production. Information on abnormal health conditions in animals obtained from these inspection data are important for farm veterinarians to detect problems in fattening management at the early stage. These data are recorded over several years for all farms that send swine to a slaughterhouse. The value of these inspection data as an animal health surveillance tool has been highlighted in several recent reports by the European Food Safety Authority [1–3]. In recent years, inspection data have been used to analyze livestock diseases in some countries, but these data have not yet been fully utilized in Japan [1, 3, 4].

Inspection data aggregated at the inspection center are time-series data and are suitable for time-series analysis. Time-series analysis using inspection data is very useful for objectively understanding the fattening process on farms. In fact, several studies have conducted time-series analyses of livestock diseases and production quantity [5–7]. Other studies have analyzed diseases of livestock in slaughterhouses using predictive models [8–11]. The study by Adachi et al. analyzed the incidence of mycobacteriosis in swine at the farm level with slaughterhouse data using autoregressive integrated moving average (ARIMA) and seasonal autoregressive integrated moving average (SARIMA) models. They also analyzed the expected monthly number of swine livers with echinococcal infection using a two-part model. Haredasht et al. modeled and predicted the rate of cattle carcass condemnation in California using dynamic harmonic regression. However, the state-space model for analyzing infectious diseases on swine farms has not yet been constructed.

The state-space model is a time-series model that uses Bayesian statistics. Because this model revises predictions using past data, it is robust against missing values and can make more accurate predictions. This model also has the advantage of being able to handle non-stationary models. Therefore, the aim of this study was to evaluate morbidity in swine using the state-space model. Using this predictive model, farmers and farm veterinarians can objectively assess animal health and better control infectious diseases.

## Results

### Model construction

The inspection data were transformed using logit transformation, and the periodicity was confirmed using correlograms. Nine models were constructed using the state-space model and R package “KFAS” (Table 1) [12]. Table 2 shows the results obtained using the auto.arima function for slaughterhouse inspection data. Predictions using the state-space model were evaluated using cross-validation, and the most appropriate model was obtained (Table 3, Additional file 1). As a result of auto.arima, models that did not include seasonality or trends were selected for numerous diseases. Using the correlogram, only mycobacteriosis and pericarditis were confirmed to have periodicity (Additional file 2). This result was consistent with the results using the auto.arima function and cross-validation (6 months). The results using the Akaike Information Criterion (AIC) and cross-validation showed a slight difference (Table 4, Additional file 1). Differences were found between evaluations using AIC and cross-validation for the five diseases interstitial hepatitis, mycoplasmal pneumonia of swine, mycobacteriosis, parasitic hepatitis, and pericarditis.

**Table 1:** Nine models constructed using the state-space model

	Seasonality	Trend
Model1	None	None
Model2	None	Linear
Model3	None	Quadratic
Model4-1	Fluctuating cycle	None
Model4-2	Fixed cycle	None
Model5-1	Fluctuating cycle	Linear
Model5-2	Fixed cycle	Linear
Model6-1	Fluctuating cycle	Quadratic
Model6-2	Fixed cycle	Quadratic

Models were classified according to nine categories based on the combination of seasonality and trend. For seasonality, models were constructed using dummy variables. Fluctuating cycle means that the seasonal component changes, and fixed cycle means that the seasonal component is fixed. For trends, we considered linear trends and quadratic trends

**Table 2:** Model parameters decided using the auto.arima function

Disease	Auto.arima	Drift	None zero mean
PA	(1,0,0)		
Diaphragmitis	(0.1.1)		○
Enteritis	(0,1,1)		○
IH	(0,1,1)	○	○
MPS	(0,1,0)		○
Mycobacteriosis	(5,0,0)		
PH	(0,1,2)		○
Pericarditis	(0,0,4)(1,1,0)		○
Perihepatitis	(0,1,4)(1,0,0)		○
Peritonitis	(0,1,1)	○	○
Pleuritis	(3,1,0)(0,0,1)		○

The table shows the order of the ARIMA model determined using auto.arima. Models with drift are circled in the Drift column, and models with non-zero mean residuals are circled in the non-zero mean column

PH, parasitic hepatitis; MPS, mycoplasmal pneumonia of swine; IH, interstitial hepatitis; PA, pulmonary abscess

**Table 3:** The most appropriate model for each disease determined using cross-validation

Disease	1 month	3 month	6 month	12 month	Adopted model
PA	2	1	1	1	1
Diaphragmitis	1	1	1	4–1	1
Enteritis	1	1	1	1	1
IH	2	2	1	4–1	2
MPS	1	1	1	1	1
Mycobacteriosis	3	3	5-1	3	3
PH	1	1	1	1	1
Pericarditis	4-2	4-2	4-2	4-1	4-2
Perihepatitis	4-1	1	1	4-1	1
Peritonitis	1	1	1	1	1
Pleuritis	1	1	1	6-1	1

The table shows the results of cross-validation. Cross-validation was performed for prediction intervals of 1 month, 3 months, 6 months, and 12 months. The most suitable model is shown in the column Adopted model. Numbers represent the model shown in Table 1

PH, parasitic hepatitis; MPS, mycoplasmal pneumonia of swine; IH, interstitial hepatitis; PA, pulmonary abscess

**Table 4:** Differences between evaluation using cross-validation and evaluation using the Akaike information criterion (AIC)

Disease	Cross-validation	AIC
PA	1	1
Diaphragmitis	1	1
Enteritis	1	1
IH	**2**	**1**
MPS	**1**	**2**
Mycobacteriosis	**3**	**1**
PH	**1**	**2**
Pericarditis	**4–2**	**1**
Perihepatitis	1	1
Peritonitis	1	1

This table shows the results of comparing cross-validation and AIC. Diseases (IH, MPS mycobacteriosis, PH) with different results are shown in bold. Model 1 was the most selected for any evaluation method

PH, parasitic hepatitis; MPS, mycoplasmal pneumonia of swine; IH, interstitial hepatitis; PA, pulmonary abscess

### Fitting to farm data

We conducted evaluation with predictive models using the inspection data of each farm based on the constructed models; the results are shown in Additional file 3. When models were constructed according to the cross-validation evaluation, the state-space model was the most suitable model for 96 diseases out of a total of 154 diseases (62.3%), including the 14 farms and 11 diseases investigated here. There were no differences between the models for 18 diseases (11.7%). Thus, the state-space model was selected for 114 diseases (74%). In particular, there was no difference between the state-space model and the ARIMA model for diaphragmitis and enteritis. However, in the evaluation using the AIC, model 1 was selected as the most suitable model for interstitial hepatitis, mycobacteriosis, and pericarditis. In consideration of this result, we assumed that model 1 was the optimum model for all diseases. When the models were evaluated, the state-space model was the most suitable model for 104 diseases (67.5%). Thus, the state-space model was selected for 122 diseases (79.2%). For some diseases, the ARIMA model using the inspection data of each farm could not evaluate diseases whereas the state-space model could evaluate diseases other than pulmonary abscess on Farm I. This was thought to be because the ARIMA model could not be used to calculate non-stationary models, so models constructed using slaughter inspection data did not match the inspection data of each farm. Additionally, it is possible to construct the ARIMA model with the state-space model, although the models were not constructed in this study [13, 14].

### Evaluating the validity of model classification

The results of cluster analysis using dynamic time warping are shown in Table 5 and Additional file 4 and 5. The gap statistics are shown in Additional file 6 and 7; the cluster classification was determined based on this result and results of the cluster.evaluation function. As a result of using the cluster.evaluation function, unlike results using the gap statistic, the number of clusters was within 1 to 3 in many diseases (Table 5). As shown in Additional file 4, there was a certain association between model selection and cluster classification. Based on the results of cluster analysis, we considered the most dominant model within the cluster to be the model of the cluster itself, and modified the model classification as shown in Additional file 8. The optimal number of clusters was 1, and the diseases in which the classifications of the models using slaughterhouse inspection data corresponded to the models using inspection data for each farm included five diseases (pulmonary abscess, enteritis, parasitic hepatitis, perihepatitis, and peritonitis) in cross-validation and five diseases (pulmonary abscess, enteritis, pericarditis, perihepatitis, and peritonitis) using the AIC (Additional file 3 and 8). Assuming that all diseases followed model 1, six diseases (pulmonary abscess, enteritis, parasitic hepatitis, pericarditis, perihepatitis, and peritonitis) had matching classifications (Additional file 8). Additionally, in interstitial hepatitis, the number of farms selected for model 4-1 (Farm C, Farm L) and those selected for model 4-2 (Farm B, Farm D) were the same, so they were merged into model 4-1. In cluster analysis, the number of farms where model 2 was selected was the same as the number of farms where model 4-1 was selected for interstitial hepatitis (Additional file 8). In the results of cross-validation using slaughterhouse data and AIC, model 1 or model 2 was selected for interstitial hepatitis, respectively. When the interstitial hepatitis model was unified to model 2, there were many farms where the ARIMA model was more accurate than the state-space model. Because the models vary greatly from farm to farm, the model in interstitial hepatitis was integrated into model 1, which is the simplest model (Additional file 3). Based on the above results, we selected model 1 for all diseases. At this time, 123 diseases (85.7%) were consistent with the results of cluster analysis.

**Table 5:** Results of cluster analysis using DTW

Disease	Optimal number of clusters	Classification			
		Optimal	1	2	3
PA	1	0.667	0.667	0.508	0.515
Diaphragmitis	2	0.777	0.467	0.777	0.735
Enteritis	3	0.558	0.600	0.513	0.542
IH	2	0.467	0.321	0.467	0.490
MPS	2	0.455	0.338	0.455	0.682
Mycobacteriosis	1	0.479	0.307	0.353	0.386
PH	1	0.528	0.600	0.528	0.481
Pericarditis	3	0.566	0.727	0.714	0.566
Perihepatitis	5	0.556	0.727	0.533	0.611
Peritonitis	4	0.581	0.783	0.550	0.661
Pleuritis	1	0.880	0.880	0.958	0.746

Optimal number of clusters is the number of clusters based on gap statistics. Results from gap statistics show that the number of clusters is within three for many diseases. Classification is the results using the cluster.evaluation function (the Optimal column is the results using the cluster.evaluation function in the optimal number of clusters). This function also indicates that the larger the value, the better the fit

PH, parasitic hepatitis; MPS, mycoplasmal pneumonia of swine; IH, interstitial hepatitis; PA, pulmonary abscess; DTW, dynamic time warping

### Depiction of graph

**Fig. 1:**
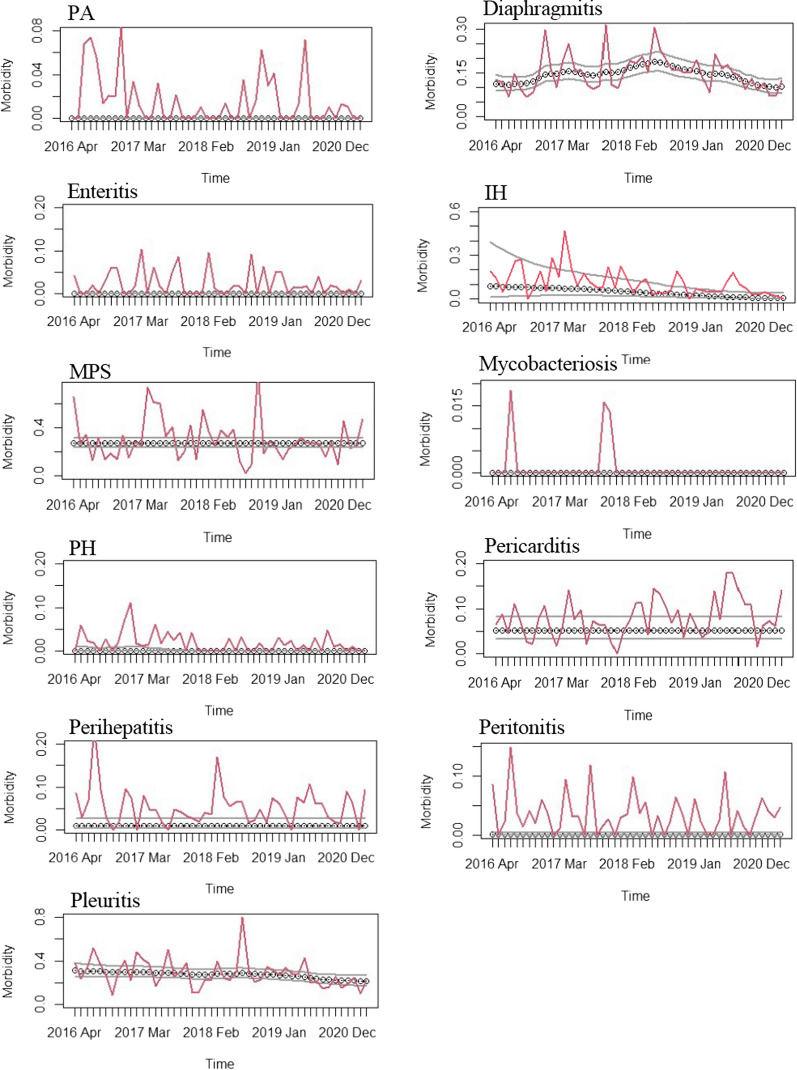
Evaluation of morbidity on Farm A. The figure shows evaluations for 11 major diseases on Farm A. The horizontal axis is time, the vertical axis is morbidity, the smoothed value is indicated by the dotted line, the 85% confidence interval is indicated by the black line, and the change in actual morbidity is indicated by the red line

Figure 1 shows the results of smoothing the past morbidity for Farm A. In the evaluation graphs, the actual morbidity may be below the smoothed value. If the actual morbidity is below the smoothed value, it is considered that the situation has improved through the efforts of the farm. However, if the actual morbidity is much higher than the smoothed value, it is considered that there may have been some problems with fattening management on the farm. Using this evaluation method, we could create simple evaluation graphs for diseases with a certain morbidity on Farm A, such as diaphragmitis and pleurisy. For diseases where the morbidity is 0% in most months, the graph was monotonic; however, we could confirm the months that deviated from 0%. However, diseases with rapidly changing morbidity, such as mycoplasmal pneumonia of swine and pericarditis, were not well characterized.

## Discussion

In this study, the state-space model was used to evaluate inspection data from a slaughterhouse. In the field of veterinary medicine, studies using predictive models have been conducted for individual diseases and rates of condemned cattle or swine, but few have analyzed farm diseases using slaughterhouse data.

In Japan, many meat inspection centers return raw data from slaughterhouses to farms, but these data include noisy data. It is difficult to obtain detailed information from raw data without analysis, and the degree of improvement in inspection results varies greatly depending on the data analysis technology of the farm to which the data are returned. Especially on farms with many elderly people, it is difficult to analyze these data, and it is not uncommon for intuitive judgments to be made using raw data. Analyzing these data can make a difference in morbidity on farms. To eliminate the inability to improve hygiene on farms where the data cannot be appropriately analyzed, a method to analyze and return data to farms is needed. However, improvement may not occur if it is difficult to interpret the results of data analysis. Therefore, it is necessary to return data in a form that is easily understood.

Time-series analysis using inspection data takes time for parameters to be set. In particular, when a meat inspection center does everything from constructing the models to the evaluation of morbidity, it is unrealistic in terms of time to choose a model for each farm at a center where many farms are evaluated. Adachi et al. also stated that it is difficult to calculate individual parameters for all farms every time [9]. In this study, detailed model classification was performed using cross-validation, the AIC, and cluster analysis, and more than half of diseases were consistent with the analysis using slaughterhouse data. To make it easier to choose a model, calculation in a realistic time frame is possible through constructing models using slaughterhouse inspection data and applying the models to the farm data. In general time-series analysis, there are deviations in estimation when this method is used. However, in the case of analysis using the state-space model, it is conceivable that the deviations in estimation are relatively small because the state is revised via Bayesian updating [15]. When data are evaluated using a general time-series model, the difference between the measured and the evaluated values becomes large in the later part of the prediction period. Such problems can be avoided by smoothing using the state-space model.

One problem in constructing time-series models is missing data. Small farms may not ship swine for months, resulting in lost data. In the state-space model, the state of missing values is revised through Bayesian updating, which offers an advantage in that there is no concern about processing these values. Because the state-space model can determine the value excluding noise data via smoothing, it is considered an effective method, especially when evaluating past diseases. In fact, the state-space model is also used for change-point and anomaly detection [16, 17]. However, there are few reports on the construction of predictive models using the state-space model in the veterinary field; it is a bit more difficult to build predictive models using this model than other predictive models. However, one study used this model to monitor the weight of pigs [18].

In our study, evaluation models for each disease were constructed from slaughterhouse inspection data. In this case, the state-space model could construct more accurate models than the ARIMA model. When constructing models for each disease, the ARIMA model could not construct evaluation models for some diseases using the inspection data of each farm. This is probably because the ARIMA model is a stationary prediction model. However, cross-validation using farm data showed no difference between the ARIMA model and state-space model for diaphragmitis and enteritis. This is because the local-level models chosen for these diseases are equivalent to the models the order chosen by auto.arima [19].

No seasonal model was selected in evaluation using the AIC in this study. Diseases in swine shipped to slaughterhouses are the result of illness at some point during the 6-month fattening period between birth and shipment. Therefore, even if a disease itself has seasonality and trends, it is unlikely to appear in the slaughterhouse inspection data. In fact, only 3 of 35 diseases were seasonal in a study using predictive models of condemned cattle [8].

In this study, dynamic time warping was used for model classification. However, dynamic time warping is a classical analysis method. In recent years, a method called derivative dynamic time warping has been proposed [20]. Additionally, one study proposed a clustering method for time-series slaughterhouse data [21]. It is important to consider how much the accuracy of classification can be improved using such methods in the future. Using dynamic time warping for analysis, it is possible to classify the model based on the features of morbidity data. However, this method also has the problem that it does not shorten the time required to create an evaluation graph because it is necessary to classify models for all farms. Considering the results of this research, it is more realistic in the field to determine the model using results of cross-validation and the AIC instead of using dynamic time warping.

In this study, the AIC and cross-validation were used for model evaluation. When performing cross-validation, the root mean square error (RMSE) was used for calculation. However, because the RMSE the difference between the measured value and the predicted value, handling of outliers tends to be strict. Therefore, it should be noted that even if the prediction is deviated owing to human factors such as the improvement of morbidity, the RMSE value will increase and the evaluation capability of the model will deteriorate.

This study has some limitations. First, 11 diseases from 14 farms were used for the analysis in this study. The data were collected at a slaughterhouse in Miyazaki Prefecture. Thus, the number of diseases, farms, and slaughterhouses was limited. Second, the model in this study was constructed based on the assumption of linearity and normality. However, given that models are less accurate for some diseases, there may be diseases with non-linear and non-normal characteristics [22]. It is necessary to verify the model for these properties in the future. In particular, for some diseases with monotonous evaluation graphs, many diseases had low morbidity; for such diseases, it is necessary to use the number of condemned swine rather than prevalence and assume non-normality. Third is the problem of using dynamic time warping. In this study, we constructed models using data features (trend and seasonality) with the state-space model. Dynamic time warping is a method that compares the similarity of graphs, and the use of dynamic time warping is not common. For this reason, it is necessary to consider that depending on the type of prediction model, it is not possible to classify the model appropriately even if it is evaluated using dynamic time warping.

## Conclusions

This study showed that it is possible to evaluate past morbidities using the state-space model. Evaluations of past disease lead to objective understanding of problems in fattening management. However, to make full use of this system, farms must know the shipping information and fattening information for lots of swine shipped to a slaughterhouse. This will help farm managers to be aware of the condition and fattening status of swine on the farm. Another important issue is the condemnation standards owing to disease at meat inspection centers. If the condemnation standards differ depending on the inspector, appropriate evaluation data cannot be obtained. Promoting related research may contribute to improving various problems on farms and in slaughterhouses that have not received sufficient attention to date but that will contribute to improvement of the livestock industry.

## Methods

### Data collection

**Fig. 2:**
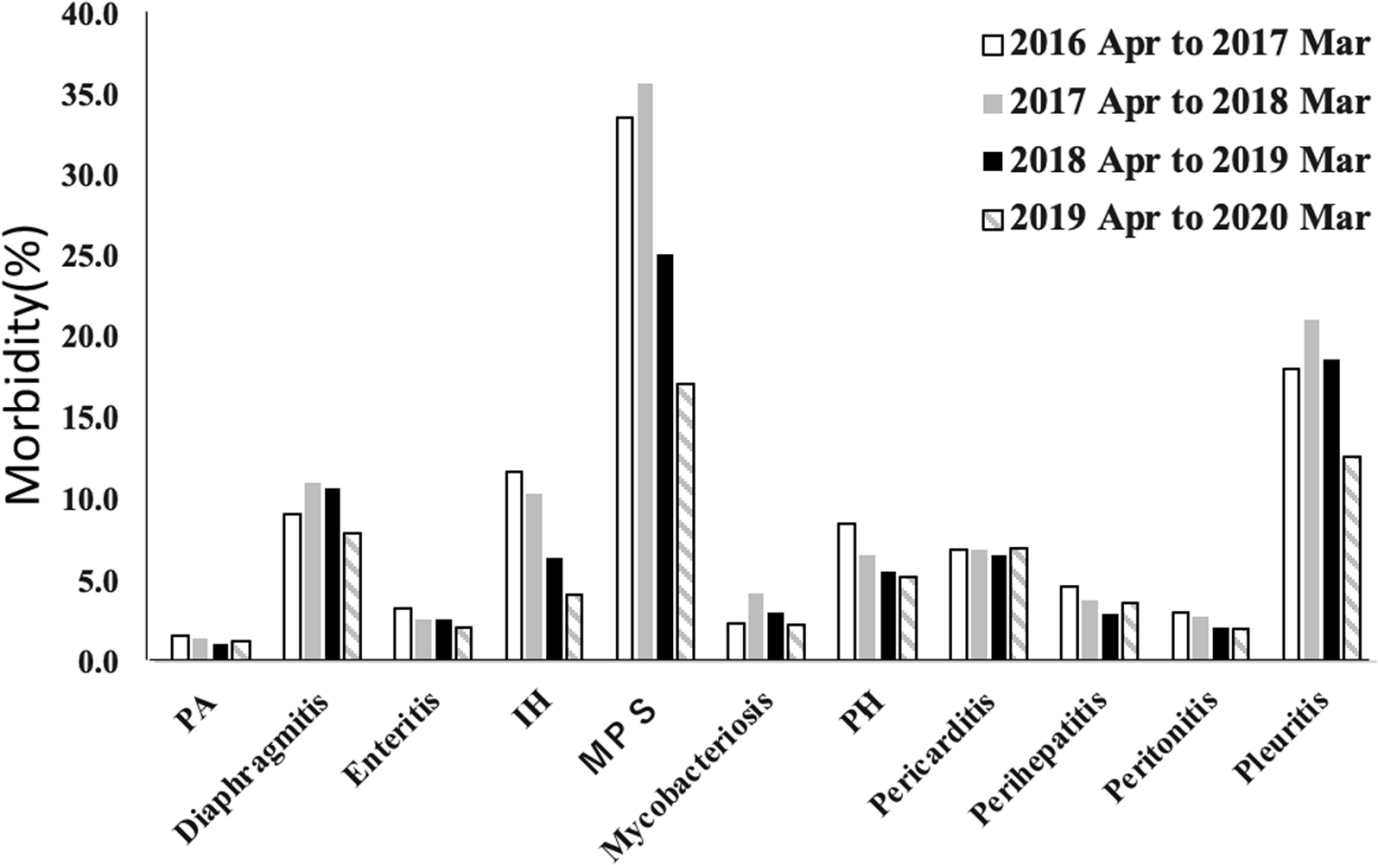
Morbidity for major 11 diseases in slaughterhouses over the past 4 years. The figure shows morbidity in the surveyed slaughterhouses over the past 4 years. The horizontal axis shows the disease name, and the vertical axis represents morbidity. Morbidity from April 2016 to March 2017 is in white, the morbidity from April 2017 to March 2018 is in grey, the morbidity from April 2018 to March 2019 is in black, and the morbidity from April 2019 to March 2020 is shown in diagonal pattern

This study was conducted in a slaughterhouse in Miyazaki Prefecture, which is located in the Kyushu region of southern Japan. Data collection was performed from April 2016 to March 2020 in the slaughterhouse. Approximately 10,000 to 20,000 pigs are slaughtered every month at this slaughterhouse, and approximately 200,000 pigs are slaughtered annually. The monthly inspection data for each farm was obtained for 11 major diseases (pulmonary abscess, diaphragmitis, enteritis, interstitial hepatitis, mycoplasmal pneumonia of swine, mycobacteriosis, parasitic hepatitis, pericarditis, perihepatitis, peritonitis, pleuritis) from the meat inspection center (Fig. 2).

The monthly morbidity was defined as

$$p_{i} = \left( {\text{condemned swine}} \right)_{i} /\left( {\text{slaughtered swine}} \right)_{i}$$

Subscript “$i$” means month (e.g., $i$ = 1 is April 2016).

### Model construction

For 11 major diseases recorded in the slaughterhouse, time-series models were constructed on the basis of slaughterhouse inspection data from all farms sending swine to the slaughterhouse. Models were constructed using the state-space model, ARIMA model, and SARIMA model [13]. In this study, all analyses were performed using R (R Core Team, 2020) [19]. Prior to the analysis, morbidity rates of the 11 diseases were transformed using the following equation (logit transformation):

$${\text{logit}}(p_{i} ) = {\text{ ln}}(p_{i} /1 - p_{i} )$$

Here, since the logit function diverges when the data value is ${p}_{i}$ = 0 or ${p}_{i}$ = 1, these values were replaced with sufficiently small or large values (${p}_{i}$ = 0.0000001 or ${p}_{i}$ = 0.9999999).

Models were constructed for each disease. For the ARIMA and SARIMA models, appropriate models were constructed for each disease using the auto.arima function [23].

### Model evaluation

To select appropriate prediction models, model evaluation was performed using the AIC and cross-validation for nine constructed models. For cross-validation, data sets were created by shifting the process of making predictions using 24-month training data by 1 month. The verification was performed using predictions 1, 3, 6 and 12 months into the future. The verification period was from April 2016 to March 2020. For the dataset, we used the RMSE to validate the most appropriate models, which is expressed using the following formula.

$${\text{RMSE }} = \sqrt {\sum\nolimits_{i = 1}^{n} {\left( {f_{i} - y_{i} } \right)^{2} /n} }.$$

where *n* is the number of data values, *f*_*i*_ is the predicted value, and *y*_*i*_ is the actual value.

### Fitting to the farm data

Predictive models that were constructed on the basis of slaughterhouse inspection data were evaluated for each disease using the inspection data for each farm. In this study, 14 farms were randomly selected from among those that brought swine to the study slaughterhouse in Miyazaki Prefecture, and predictive models were evaluated for those farms. The prediction period of prediction models to be evaluated was 1 month. For model evaluation, two models, the state-space model and an ARIMA and SARIMA model were compared. Assuming that the same disease has the same trend and seasonality, state-space models on farms were constructed on the basis of the cross-validation and AIC results obtained using the slaughterhouse inspection data. For ARIMA models, we constructed predictive models based on the results of applying auto.arima to the slaughterhouse inspection data. For each model constructed, the models were verified by performing cross-validation using the inspection data for each farm.

### Evaluating the validity of model classification

Predictive models constructed on the basis of the slaughterhouse data do not always match those constructed on the basis of farm data. Because the state-space model classifies models based on the properties of graphs such as seasonality and trend, in this study, it was assumed that there are relationships between the shape of the graphs and model classifications, and the validity of the model classification was evaluated on the basis of the shape of graphs. Therefore, with hierarchical clustering using dynamic time warping, the appropriate number of models for each disease was determined on the basis of the shape of the time-series graph [24]. DTW is a method for determining the similarity of time series graphs. When there are two time series data $x$ and $y$, let $w$ = (${w}^{x}$, ${w}^{y}$) be that time $t$ = ${w}^{x}$ of waveform $x$ and time $t$ = ${w}^{y}$ of waveform $y$ match due to alignment., and the entire waveform alignment is represented by a set of $K$ pieces $W$.

$$W = \left\{ {w_{1} ,w_{2} , \ldots w_{K} } \right\}$$

At this time, the distance between waveforms by DTW is defined as follows using Euclidean distance.

$${\text{DTW}}(x,y) = \mathop {\min }\limits_{{\phantom{i}}} \mathop \sum \limits_{K}^{k} | x_{{w_{k}^{x} }} - y_{{w_{k}^{y} }} |$$

For the time-series graph, morbidity data of 14 farms were used. The “TSclust” package was used for the calculation of dynamic time warping [25]. To examine the number of models, the “cluster” package was used to calculate the gap statistic [26]. The number of clusters is the minimum $k$ that satisfies the following equation:

$${\text{Gap}}(k) \ge {\text{Gap}}(k + 1) - {\text{SE}}.{\text{sim}}(k + 1)$$

The minimum $k$ for which the gap statistic satisfies the above formula was defined as “The optimal number of clusters”.

After calculating the gap statistic, the correct classification rate was calculated using the cluster.evaluation function. The correct classification rate was calculated for the number of clusters from 1 to 3 and the largest gap statistic, and the number of clusters with the highest correct classification rate was defined as the modified number of clusters.

### Depiction of graph

Based on the constructed predictive models, evaluation graphs of the past morbidity rate for each farm were drawn for each disease. In drawing the evaluation graphs, the logit transformed value was inversely converted using the following formula.

$$p_{i} \left( x \right) = e^{x} /\left( {1 + e^{x} } \right)$$

The confidence interval for graphs was set to 85%.

## Supplementary Information

**Additional file 1**. Results of Cross-validation and Akaike Information Criterion (AIC) on slaughterhouse data. PH, parasitic hepatitis; MPS, mycoplasmal pneumonia of swine; IH, interstitial hepatitis; PA, pulmonary abscess; AIC, Akaike Information Criterion.

**Additional file 2**. Correlogram for each disease. PH, parasitic hepatitis; MPS, mycoplasmal pneumonia of swine; IH, interstitial hepatitis; PA, pulmonary abscess.

**Additional file 3**. Evaluation results of models. PH, parasitic hepatitis; MPS, mycoplasmal pneumonia of swine; IH, interstitial hepatitis; PA, pulmonary abscess; AIC, Akaike Information Criterion; ARIMA, autoregressive integrated moving average; RMSE, root mean square error.

**Additional file 4**. Model classification by dynamic time warping (DTW). PH, parasitic hepatitis; MPS, mycoplasmal pneumonia of swine; IH, interstitial hepatitis; PA, pulmonary abscess.

**Additional file 5**. Clustering of farms (for each disease) using dynamic time warping (DTW).

**Additional file 6**. Gap statistics for each disease. PH, parasitic hepatitis; MPS, mycoplasmal pneumonia of swine; IH, interstitial hepatitis; PA, pulmonary abscess.

**Additional file 7**. Results of the gap statics. PH, parasitic hepatitis; MPS, mycoplasmal pneumonia of swine; IH, interstitial hepatitis; PA, pulmonary abscess; k, the number of cluster.

**Additional file 8**. Revised cluster classification. PH, parasitic hepatitis; MPS, mycoplasmal pneumonia of swine; IH, interstitial hepatitis; PA, pulmonary abscess; DTW, dynamic time warping.

## Data Availability

Data related to this study can be made accessible by request to the corresponding author.
