# Supplementary material for: Evaluating swine disease occurrence on farms using the state-space model based on meat inspection data: a time-series analysis
Source: Porcine Health Manag. 2024 Jan 23;10:6. doi: 10.1186/s40813-024-00355-z (PMC11378582; doi:10.1186/s40813-024-00355-z)
Supplement: Supplementary file 2 — Additional file 2. Correlogram for each disease. PH, parasitic hepatitis; MPS, mycoplasmal pneumonia of swine; IH, interstitial hepatitis; PA, pulmonary abscess. [file 40813_2024_355_MOESM2_ESM.pdf]

# Supplementary Data 2：The correlogram for each diseases

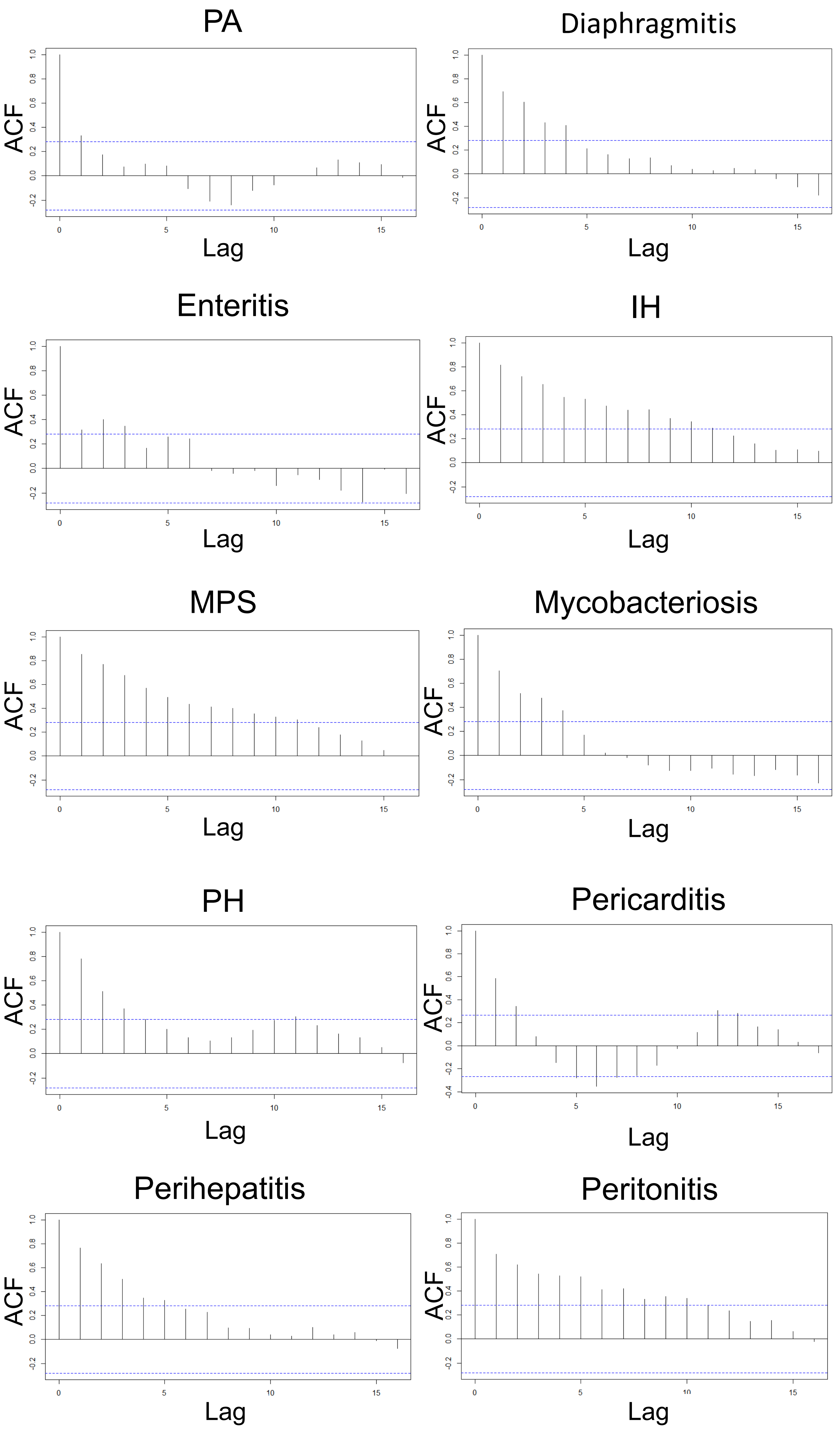

Pleuritis

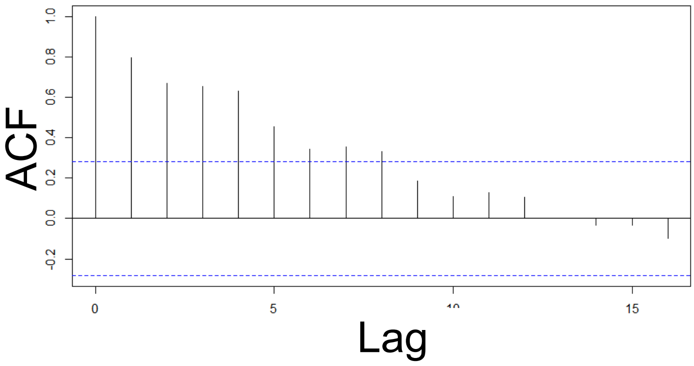


The figure shows the correlogram for each disease using inspection data of a slaughterhouse. In each graph, the vertical axis indicates ACF(Autocorrelation Function), and the horizontal axis indicates lag.

Narita et al.
