# Supplementary material for: Evaluating swine disease occurrence on farms using the state-space model based on meat inspection data: a time-series analysis
Source: Porcine Health Manag. 2024 Jan 23;10:6. doi: 10.1186/s40813-024-00355-z (PMC11378582; doi:10.1186/s40813-024-00355-z)
Supplement: Supplementary file 5 — Additional file 5. Clustering of farms (for each disease) using dynamic time warping (DTW). [file 40813_2024_355_MOESM5_ESM.pdf]

Supplementary data 5: Clustering of farms (for each disease) using dynamic time warping (DTW)

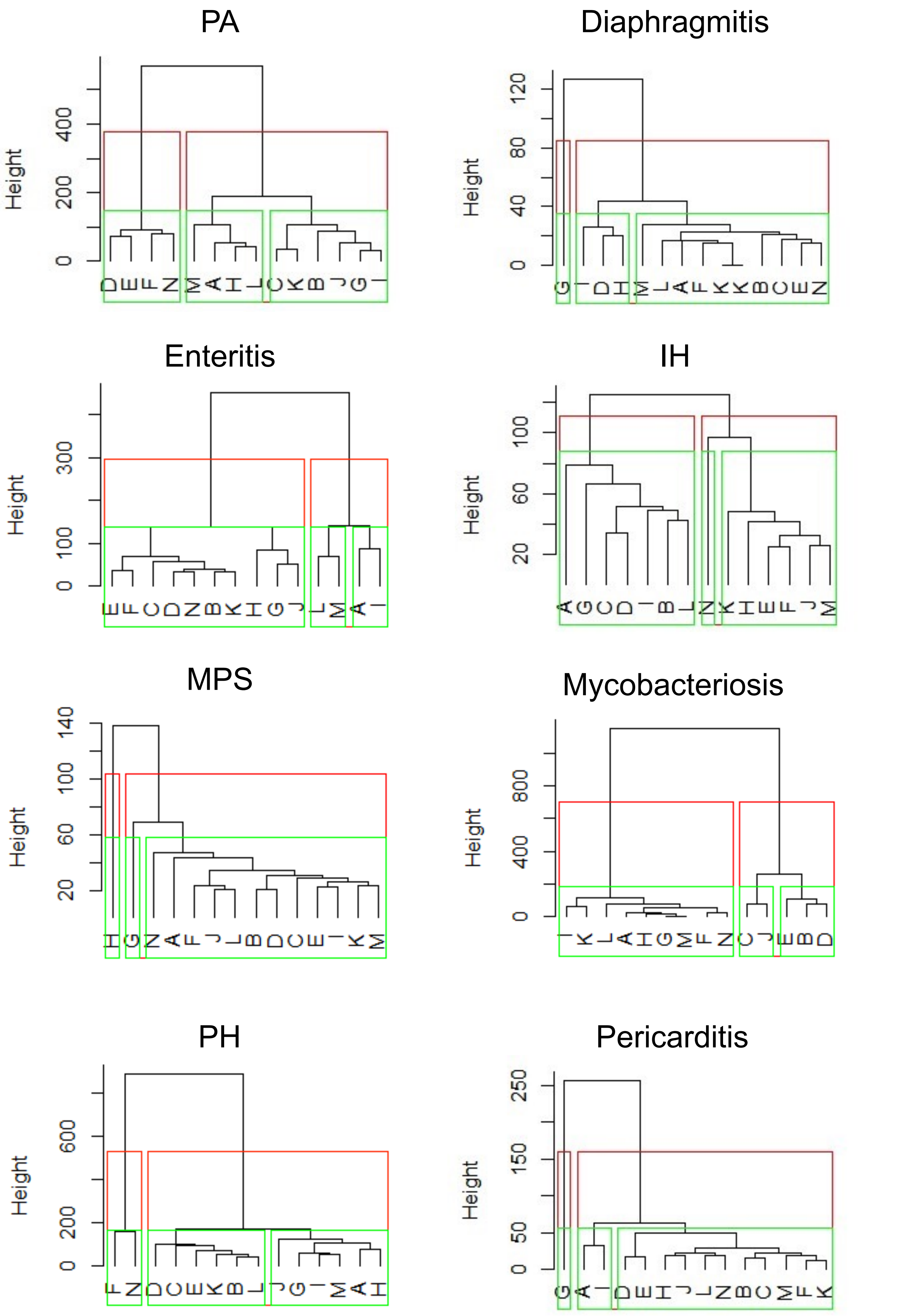

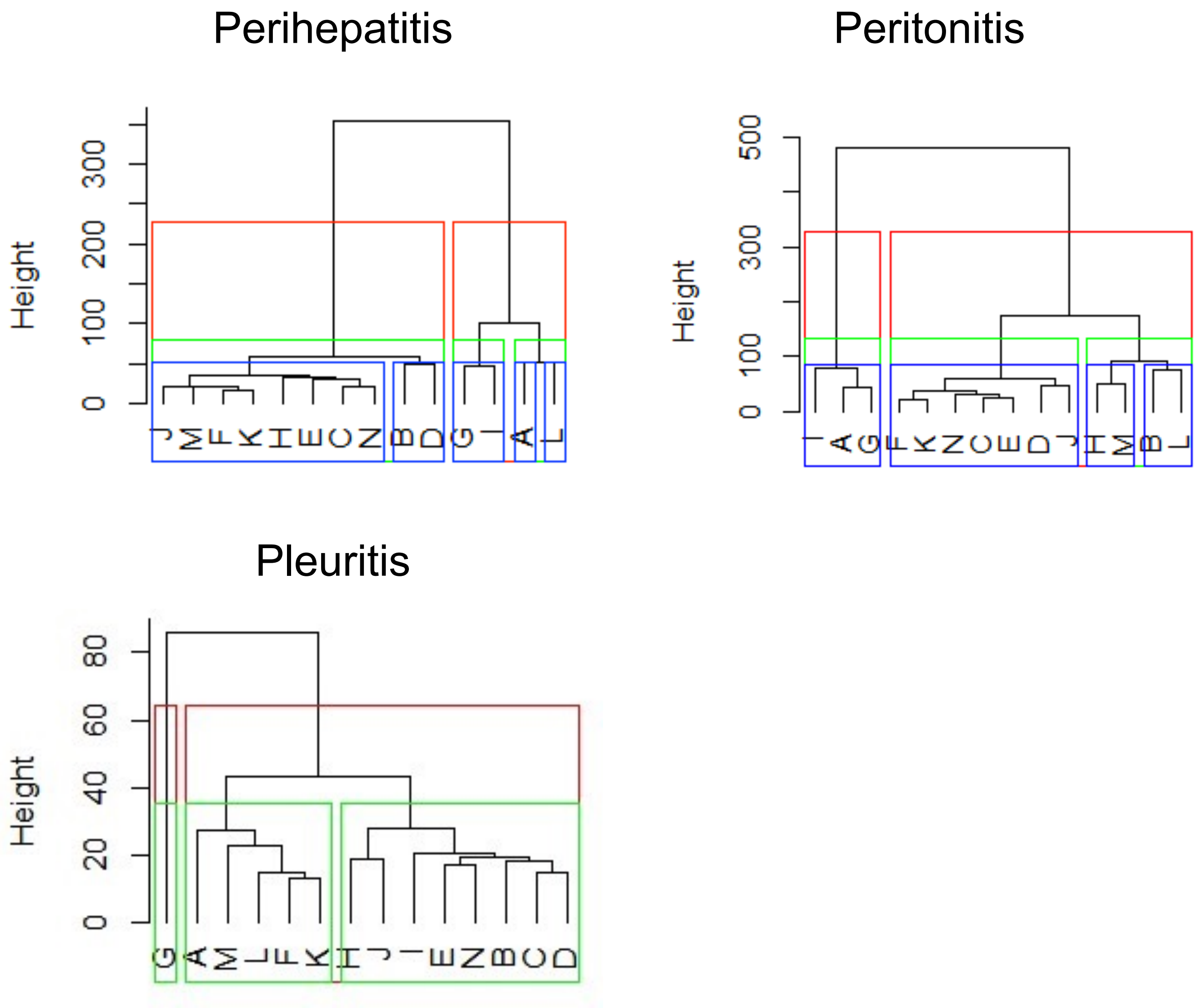

The figure shows the dendrogram of farm clustering by DTW. A to N on the horizontal axis indicate farms, and the vertical axis indicates distance. Red line indicates classification by 2clusters, green line Indicates classification by 3clusters, and blue line indicates classification when the optimal number of clusters is 4 or more.
