# Supplementary material for: Evaluating swine disease occurrence on farms using the state-space model based on meat inspection data: a time-series analysis
Source: Porcine Health Manag. 2024 Jan 23;10:6. doi: 10.1186/s40813-024-00355-z (PMC11378582; doi:10.1186/s40813-024-00355-z)
Supplement: Supplementary file 6 — Additional file 6. Gap statistics for each disease. PH, parasitic hepatitis; MPS, mycoplasmal pneumonia of swine; IH, interstitial hepatitis; PA, pulmonary abscess. [file 40813_2024_355_MOESM6_ESM.pdf]

Supplementary Data 6：Gap statistics for each disease

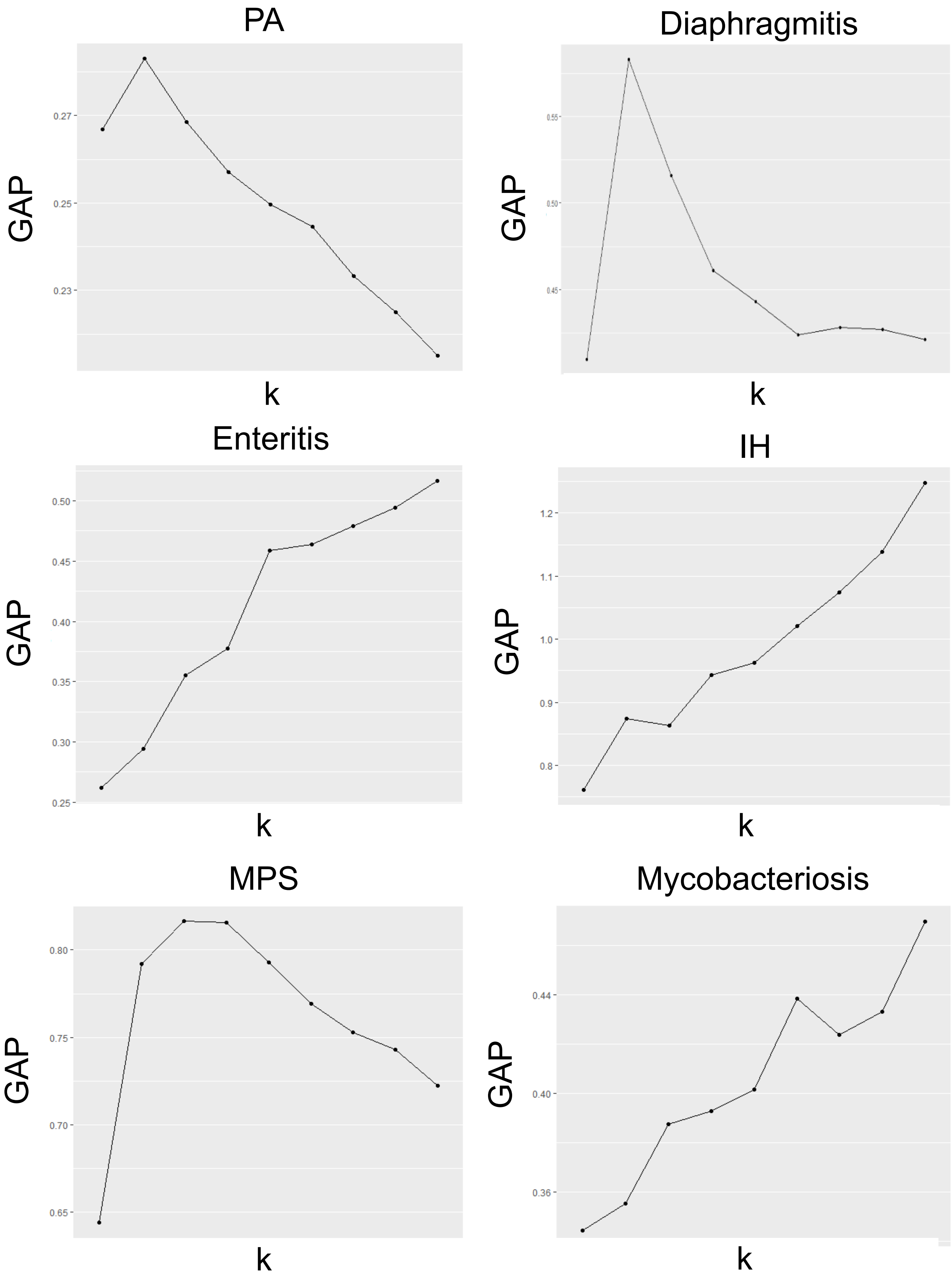

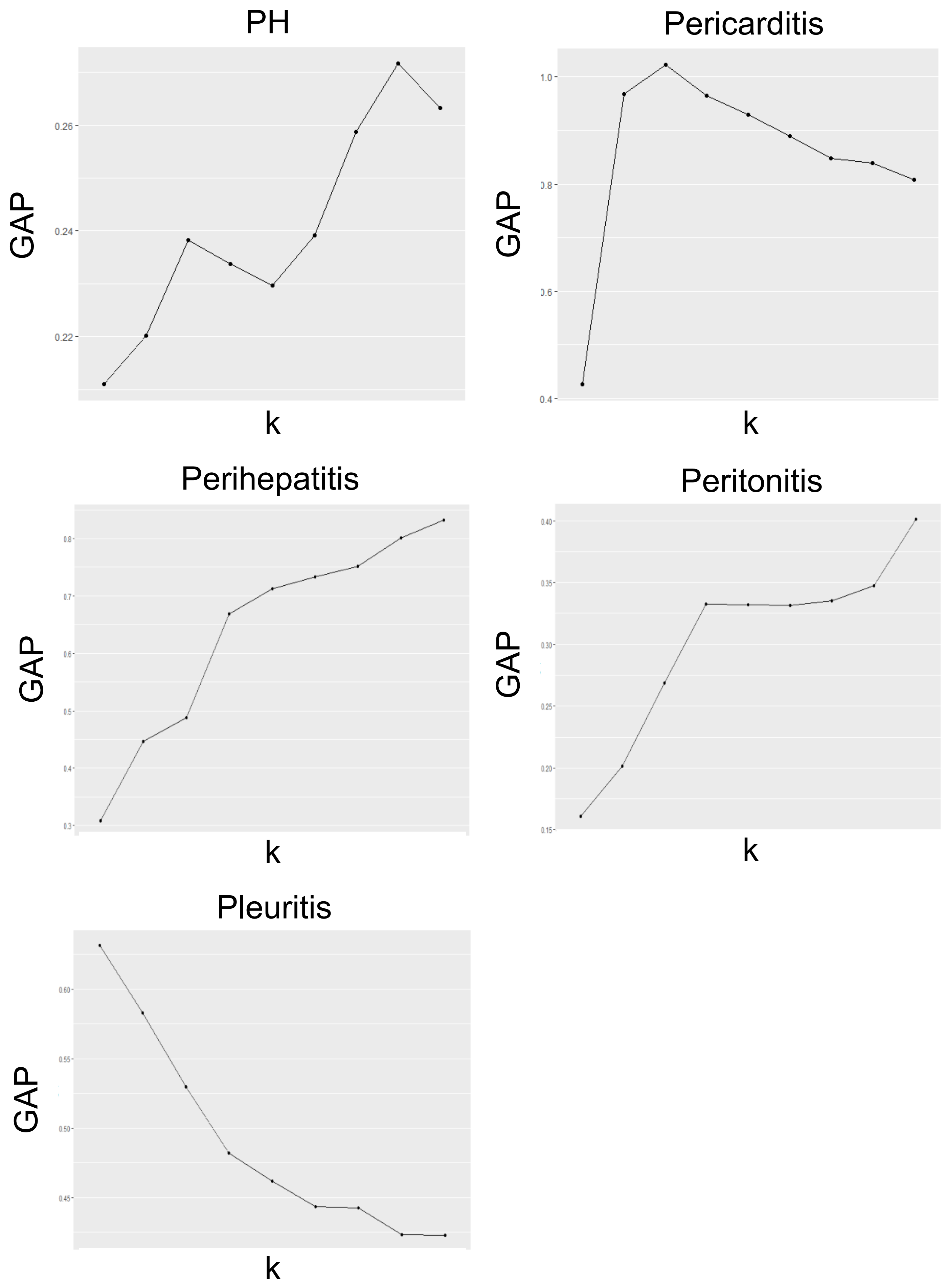

The figure shows the gap statistics for each disease. In the figure, the vertical axis indicates gap statistics, and the horizontal axis k indicates the number of clusters.

Narita et al.
